# Adverse commercial determinants of health in low- and middle-income countries: a public health challenge

**DOI:** 10.1093/heapro/daaf193

**Published:** 2025-11-18

**Authors:** M Mofizul Islam, Cassandra De Lacy-Vawdon, Deborah Gleeson

**Affiliations:** Department of Public Health, La Trobe University, Bundoora, Melbourne, Victoria 3086, Australia; Department of Public Health, La Trobe University, Bundoora, Melbourne, Victoria 3086, Australia; Department of Public Health, La Trobe University, Bundoora, Melbourne, Victoria 3086, Australia

**Keywords:** Commercial determinants of health, low- and middle-income countries, profit motive, public health, commercial practice, small- and medium-sized enterprises

## Abstract

Research on the Commercial Determinants of Health (CDoH) has primarily focused on high-income countries and large commercial entities, with limited scholarly literature addressing the practices of smaller commercial entities in low- and middle-income countries (LMICs). However, LMICs face the greatest public health challenges resulting from harmful commercial activities, and most commercial entities in these countries are small and medium-sized enterprises (SMEs). This perspective article examines harmful practices commonly adopted by SMEs in LMICs, drawing on a published framework of commercial sector practices and portfolio. By sharing examples of these practices, the paper highlights harmful actions of SMEs in LMICs, illustrates the significant public health burden they create, and calls for more research and policy focus on these issues. There is an urgent need for CDoH research in LMIC contexts, which should include documenting and monitoring the activities of commercial actors, including SMEs. It is essential for researchers from LMICs to be involved in this research, and efforts should focus on building capacity in this area. To minimize the harms associated with these commercial practices, local regulatory action is required, alongside support from intergovernmental organizations such as the World Health Organization.

Contribution to Health Promotion

- There is little research about commercial determinants of health (CDoH) in low- and middle-income countries (LMICs).
- In LMICs, small and medium enterprises (SMEs) are critical in creating both beneficial and adverse health outcomes.
- Although the practices and harms related to CDoH occur in both developing and developed countries, the public health burden is heavier in LMICs due to a lack of effective regulation, limited resources, and the need to secure basic services and pursue economic development.
- Harmful commercial practices involving SMEs are widespread in LMICs. This study highlights the urgent need for further research and policy action in this area.

## INTRODUCTION

The commercial determinants of health (CDoH) are now understood as ‘the systems, practices, and pathways through which commercial actors drive health and equity’ (Gilmore *et al.* 2023) or ‘a key social determinant, [referring] to the conditions, actions and omissions by commercial actors that affect health’ (World Health Organization 2023). Key to these framings is the understanding that CDoH can have beneficial and/or detrimental impacts on health that are complex and can co-occur (Gilmore *et al.* 2023, World Health Organization 2023). The academic origin of the concept of CDoH is linked with political economy approaches and the literature on this topic aims to mitigate or reduce the health harms caused mainly by large commercial actors and related entities and/or to enhance benefits associated with commercial entities (Anaf *et al.* 2020). CDoH can positively impact health and well-being by creating beneficial or essential goods and services, as well as generating conditions that support health, such as employment opportunities (Gilmore *et al.* 2023). However, they can also lead to negative health outcomes, which have so far received the most attention from public health scholars. Discussions surrounding CDoH in public health frequently refer to the alcohol, tobacco, and ultra-processed food industries as major examples (de Lacy-Vawdon and Livingstone 2020, Burgess *et al.* 2024). Recently, other sectors such as gambling, firearms, automobiles, and mining have also started to receive attention (Douglas *et al.* 2011, Millar 2013, Maani *et al.* 2020, van Schalkwyk *et al.* 2021, Friel 2023).

Over the past decade, there has been an increasing awareness of the CDoH and their impact on population health. However, there is limited literature available on this topic in the context of low- and middle-income countries (LMICs). A recent systematic review by Burgess et al (2024) highlighted the minimal research on CDoH specifically in LMICs, noting that among the articles with a specific regional focus, most concentrated on high- and middle-income countries, although this review may not have captured all relevant papers due to the use of search terms such as ‘corporate/commercial determinants of health’ or ‘corporate political activity’. Some studies may have examined harmful commercial practices or portfolios without explicitly using these key terms. It is also worth noting that the review was conducted in 2022, and there may have been further relevant studies published since this time.

LMICs have recorded increased consumption of soft drinks, processed foods, tobacco, and alcohol at a faster pace than has occurred historically in high-income countries (Stuckler *et al.* 2012). These changes are often part of a concerted effort by large commercial entities to expand their business and customer base within LMICs. As the scale and effects of these unhealthy products are large, they often attract public health attention. This may explain why much of the CDoH literature focuses on multinational and large commercial enterprises. While this focus is understandable and justified, in LMICs, small and medium enterprises (SMEs) also play a critical role in creating both beneficial and adverse health outcomes. Indeed, in LMICs, SMEs often serve as the backbone of the economies (World Bank Group 2019). Thus, a focus on these local businesses may bring greater granularity to our understanding of CDoH within LMIC contexts.

This paper aims to explore the commercial practices of SMEs in low- and middle-income countries that may adversely affect population health. We use a framework of commercial practices, drawing on the published literature for examples. We also discuss the importance of addressing SME commercial practices alongside the practices of larger commercial entities.

## COMMON CDoH ASSOCIATED WITH COMMERCIAL SECTOR PRACTICES WITHIN LMICs

Commercial environments in LMICs share some similarities with those in high-income countries, but they also possess important differences. So far, these differences have not been systematically explored in the context of the CDoH literature. In his section, we aim to address this gap by applying the CDoH model proposed by Gilmore et al (2023) and Lacy-Nichols et al (2023), specifically focusing on CDoH practices and portfolio to examine key examples of commercial sector practices within LMICs. We will present some case examples of common commercial practices by SMEs in LMICs that have not been adequately discussed in the CDoH literature. While some of these examples occur in other settings, they are more common in LMICs and are likely to have severe effects in these regions. A general overview and definition of commercial practices, along with their examples in LMIC contexts, are provided in Table 1. These examples will be discussed in greater detail throughout the rest of this section, followed by an examination of their implications for the broader literature in the discussion section of the paper.

**Table 1.** Commercial sector practices and portfolio and examples from small- and medium-sized commercial entities in LMICs.

Practices	Definition	Examples of practices involving SMEs within LMICs
Political	Practices to secure preferential treatment, prevent or favourably shape policies, and circumvent or undermine policies.	Bribing of government officials by commercial entities in public works and construction sectors.
Scientific	Practices involving the production and use of science to alter products or otherwise secure industry-favourable outcomes, or both.	Deception, fraud and data falsification in the pharmaceutical industry; employing free market thinktanks to convince the government against tobacco tax increases.
Marketing	Practices to promote sales of products or services.	Aggressive marketing by pharmaceutical companies and care providers leads to undue use that causes harm.
Supply chain and waste	Practices involved in the creation, distribution, retail and waste management of products or services.	Market syndicates and associated artificial price hikes for food and other essential products. Hazardous industrial waste disposal and excessive use of pesticides.
Labour and employment	Practices to manage those employed directly within or under contract to the organisation within its supply chain	Unsafe workplaces in apparel factories and inadequate labour laws for compensation.
Financial	Practices to support the financial position of the organisation.	Syphoning foreign currencies outside of the country by inappropriate banking systems, stock market manipulation, and tax evasion. This then causes the erosion of public organisations e.g. inadequate foreign currencies to purchase essentials from other countries.
Reputational management	Efforts to shape legitimacy and credibility, reduce risk, and enhance corporate brand image.	Corporate social responsibility efforts of alcohol and tobacco industries, e.g. donation of a large amount of money to help the Thai government’s 2004 tsunami relief efforts,
Portfolio of products	Range and types of goods and services produced.	Production of items that are harmful for health, e.g. adulterated food.

Definitions adapted from Lacy-Nichols et al. (2023).

The illustrative case examples were selected based on the authors’ familiarity with how SMEs in LMICs, directly or indirectly, engaged in seven commercial practices and the additional product portfolio category outlined in Table 1. This paper uses this framework as a lens to explore and discuss how a subset of SMEs can also contribute to adverse health outcomes in LMICs. We started with a set of case examples, mapped these to the eight categories in the commercial practice and portfolio framework, and, where necessary, identified selected additional case examples to ensure coverage of all eight categories. All data used were publicly available and ethics approval was not sought for this study.

## POLITICAL PRACTICES

The political practices of SMEs with implications for health vary from lobbying, political donations and funding social programmes to bribery (Gilmore *et al.* 2023). SMEs can engage in government consultations and work collectively, sometimes alongside large corporations, potentially influencing policy, bending regulations, and taking advantage of poor governance and corrupt political systems (Harstad and Svensson 2011, Abdool Karim *et al.* 2020). Relatively weak governance systems in LMICs and/or inadequate enforcement of existing regulations may exacerbate this situation (Abdalla *et al.* 2023, World Bank Group 2025). Bribing political leaders, influential people, and government officials appears to be a common approach for SMEs. For instance, bribery is an open secret in the public works sector in many LMICs. Individuals, construction farms, and public works organizations pay bribes to government officials for the completion approval of low-quality or faulty infrastructure projects, such as bridges, roads, or buildings. These substandard constructions can result in significant public health hazards and fatalities (Kenny 2007). Poorly constructed roads, improperly built sanitation systems and unsafe housing can result in death and/or long-term health risks, such as increased accidents, exposure to environmental toxins and the spread of disease. Contractors, who are often local businessmen, use these practices to maximize profits and avoid regulatory requirements. For instance, an investigation into the Rana Plaza collapse in Bangladesh, which killed more than 1100 garment workers, found that it was built based on illegal construction codes (Goodwin 2021). Also, solely for commercial gain, the owner had added several floors without obtaining the necessary permits. Despite visible cracks in the walls that had raised safety concerns among workers the day before the collapse, management insisted that they enter the building (Westby 2014). Although corruption exists in all countries, it is more prevalent in LMICs (Transparency International 2024) and corruption in public infrastructure is particularly nefarious for LMICs (Hawkesworth 2020). Estimates of losses to bribery in the construction sector are as high as 45% of construction costs in LMICs.

## SCIENTIFIC PRACTICES

Scientific practices aim to shape the environment of evidence and knowledge to achieve favourable outcomes for commercial entities (Gilmore *et al.* 2023). The commercial sector seeks to influence scientific process to arrive at conclusions that support its positions and, ultimately, revenue generation and profit-making. In LMICs, where regulatory oversight is often weak, SMEs may fund or commission evidence generation that influences health regulations at local or regional levels (Kadandale *et al.* 2019). Various strategies are employed to influence scientific evidence generation, including providing funding, engaging in deception and fraud, selective reporting, and strategically choosing clinical trial locations (Legg *et al.* 2021). LMICs are often seen as attractive sites for evidence generation due to reduced expenses and less stringent regulations. For example, a randomized controlled trial on the formula supplementation of breastfed newborns for 30 days, starting within 6 hours of birth, was conducted in Uganda and Guinea-Bissau (Ginsburg *et al.* 2022). This trial raised several ethical concerns and conflicted directly with international public health recommendations regarding breastfeeding (Doherty *et al.* 2022). The famous book *Bottle of Lies: The Inside Story of the Generic Drug Boom* describes chilling accounts of deception, fraud, and data falsification in the pharmaceutical industry (Eban 2019). The book details the shipment of medicines that did not meet required standards to less rigorous markets. It is framed by the prologue and epilogue of a former Ranbaxy employee who became a notorious whistleblower, exposing the inadequate manufacturing and operating standards of Indian generic drug companies.

Arguing against public health policies using selective information is another scientific practice frequently employed in LMICs, often due to their relatively weak regulatory frameworks and oversight. In some LMICs, commercial entities collaborate with thinktanks that are influential and well-connected with top policymakers. For instance, in Malaysia, a free-market thinktank argued against tobacco tax increases, taking the position of tobacco companies by claiming that tax hikes would lead to increased tobacco smuggling and exaggerating the connection between illicit trade and tobacco taxes (Glenza 2019). The International Tax and Investment Center has, at various times, published reports and hosted forums portraying the tobacco industry as a significant contributor to government revenue, job creation, and overall economic activity in developing countries. Such tactics have proved successful in influencing tax policies in some countries (Tobacco Control Research Group 2020). The portrayal of these critical economic aspects convinces many LMICs to facilitate tobacco industries while overlooking the harmful effects of tobacco (Islam 2024).

## MARKETING PRACTICES

Marketing practices drive greater demand for and consumption of unhealthy products (Gilmore *et al.* 2023). SMEs can follow similar marketing practices to large transnational corporations, or utilise locally adapted marketing practices with high cultural salience, to increase the sales of their products. The exponential spread of access to digital media in LMICs has been heavily exploited by companies (transnational and local alike) to market unhealthy products to new audiences, including children and adolescents (Theodore *et al.* 2021). Some of the marketing approaches by SMEs involved in health-harming products may have considerable impact. For instance, in some LMICs, physicians, particularly in private clinics and community pharmacies, receive kickbacks or financial incentives from pharmaceutical companies for prescribing or selling certain drugs (Khan *et al.* 2023). This financial incentive can drive them to prescribe medications or sell over-the-counter drugs that may be unnecessary. It has been estimated that the Indian pharmaceutical industry spent approximately 342 billion rupees (nearly 4.5 billion USD) on marketing between the 2008–2009 and 2016–2017 fiscal years (D'souza 2022). Most pharmaceutical companies in LMICs are SMEs. In India, for example, about 70% of pharmaceutical entities can be classified as SMEs (India Brand Equity Foundation 2023). Some pharmaceutical companies invest twice as much in marketing and sales as in research and development (Gul *et al.* 2021) and try to induce demand for medicines via physicians, community pharmacies, and patients. This profit motive results in excessive and inappropriate use of medicines and increased polypharmacy, which can result in adverse health outcomes.

The promotion of unnecessary caesarean deliveries exemplifies another instance of commercially motivated healthcare, which can have serious adverse health effects. In many LMICs, women choosing to deliver in private facilities have over four times the odds of undergoing a caesarean section compared to those delivering in public facilities (Bhatia *et al.* 2020). This substantial difference between public and private facilities cannot be explained by known determinants of caesarean rates such as medical indications and is driven by profit motives. Some private health facilities hire agents, who act as individual brokers, to persuade patients to opt for a caesarean delivery. These agents receive financial incentives from the private healthcare system in return for their efforts. Indeed, the marketing of caesarean deliveries has become aggressive; for example, some public hospitals have reported that agents often try to convince patients to request a discharge from the public hospital so they can be transferred to a private facility for the procedure (Aminu *et al.* 2014). The list of commercially motivated services in the health sector is extensive and includes unnecessary diagnostic tests and referrals to private hospitals and providers. This healthcare provider-induced care, influenced by marketing practices, results in unnecessary costs for patients, placing a significant burden on already economically disadvantaged people in LMICs. Due to inadequate regulation and a lack of accountability (WHO 2024c), LMICs are particularly vulnerable to these marketing practices.

## SUPPLY CHAIN AND WASTE PRACTICES

While there are numerous examples of transnational corporations adopting supply chain and waste practices that negatively affect human and planetary health (Gilmore *et al.* 2023), the role of SMEs should not be overlooked. Supply chain fraud or opportunism is not uncommon in LMICs that primarily aim to increase profit margins. Hoarding of essential food items and the market syndicates in some LMICs are good examples (Silvestre *et al.* 2018, Sabur *et al.* 2021). Colluding with political actors, middlemen exploit these conditions by stockpiling commodities to create artificial scarcity (Sharma and College 2025). A notable case is the recurrent onion price crises in India, where traders and intermediaries deliberately hoard onions to restrict supply and then gradually release them to benefit from rising prices (Sharma and College 2025). Similar supply chain manipulation by middlemen is also known in Kenya and Israel (Levi *et al.* 2021).

A syndicate, in this context, refers to a group of individuals, organizations or companies that collaborate secretly to manipulate markets for their mutual advantage (Hosain 2022). Syndication commonly involves collusion, wherein business entities agree to fix prices, control the supply of goods, or create artificial shortages to maximize profits. Mainly retailers and/or wholesalers are involved in this inappropriate practice (Hosain 2022). Since most businesses in LMICs are SMEs (World Bank Group 2019), they are likely the entities involved in hoarding. Such profits are undue because they are obtained through manipulation rather than through legitimate business practices or by providing better products or services. As a result, syndicates may increase the prices of daily essentials, causing significant hardships for individuals with low incomes and leading to inadequate food and nutrition (Sarkar *et al.* 2009).

**Figure 1.**
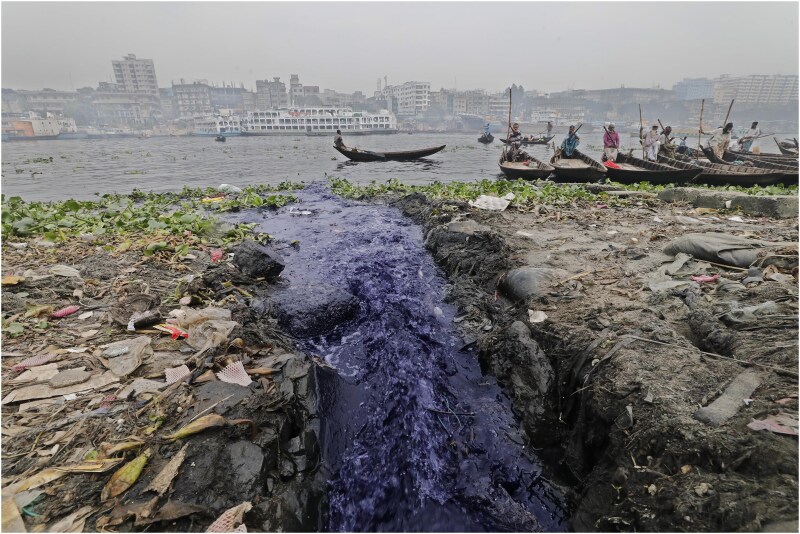
Industrial effluent enters a major river in Dhaka, Bangladesh (Photo: Kamrul Islam Ratan, Photojournalist, The Daily Sun).

In many LMICs, waste materials are disposed of directly into rivers or open fields (Ferronato and Torretta 2019) (see Fig. 1). This practice leads to severe water pollution and environmental damage, particularly in areas with open dumping sites near waterways. Rain and wind can easily carry garbage into the rivers from these sites. The unchecked disposal of waste results in significant environmental harm, including water pollution, the destruction of aquatic ecosystems, and the spread of diseases (Uddin and Jeong 2021). Additionally, it poses long-term health risks for local communities that use these water sources for drinking, bathing and agriculture. Establishing and maintaining waste management systems—including waste collection, transportation, and disposal—are costly. Even where government regulations exist, commercial entities often disregard them, prioritizing economic gain over substantial health issues for residents in those areas (Sudarshan 2022). A substantial proportion of these commercial entities are small or medium-sized businesses.

Over the last decade, global pesticide use grew 20% by volume; however, the use in low-income countries grew by 153% over the same period (Shattuck *et al.* 2023). Across LMICs, the use of pesticides substantially reduced freshwater fish populations (Ray and Shaju 2023) and the availability of affordable food for low-income communities. The rapid depletion of wild-caught fish prompted intensive aquaculture and fish farming (FAO 2022). A considerable proportion of these fish farms to increase production and reduce losses use antibiotics and non-organic chemicals (Islam 2021). The residues of these materials can accumulate in the fish themselves, which, when consumed by humans, can cause various health issues (Pelic *et al.* 2024). Also, marketing strategies by pesticide companies might influence farmers’ decisions, encouraging more use than necessary (Scholin *et al.* 2025) and all sizes of commercial entities are involved in that. There are at least two sides of this CDoH: (a) the benefits from pesticides through increasing or sustaining food production and (b) the health harms from direct exposure to pesticides and through the environment, for instance, diminishing freshwater aquatic animals and organisms. This is a classic example of the complexity of both beneficial and harmful outcomes of CDoH. In developed countries, it may be feasible to ensure an optimal use of these chemicals. However, in LMICs, there are numerous challenges in the regulatory environment, and the dire necessity of increasing food production may be prioritized.

## LABOUR AND EMPLOYMENT

LMICs generally lack access to basic labour protections like minimum wage laws, occupational health and safety regulations, or the ability to unionize and advocate for better conditions (Kavanagh *et al.* 2024). This leads to workers facing long hours, hazardous work environments, and the risk of injury or illness without compensation or support. In sectors like construction, garments, or agriculture, workers may be exposed to toxic chemicals, unsafe machinery, or inadequate sanitation, all of which pose significant health risks (Kabir *et al.* 2019). Workers may take on these dangerous jobs due to limited opportunities or poverty. Commercial entities, thereby, exploit workers’ vulnerabilities, prioritizing profit over their well-being. There are numerous instances of health harm caused by poor conditions of labour and employment in LMICs. The Rana Plaza, mentioned in the political practices section above, housed production units for several leading global fashion brands. At the time of the Rana Plaza tragedy, compensation law for workers was weak and widely unenforced (Prentice 2018). In LMICs, this issue is prevalent across all types of businesses, whether local or international, small or large. Some commercial entities may produce and market commodities that are perceived as healthy yet engage in business practices that can be detrimental to overall well-being, such as neglecting tax obligations or mistreating their workers (Mialon 2020). While these issues are present in both developing and developed countries, the public health burden is significantly greater in LMICs due to insufficient labour rights, inadequate regulations and enforcement (Courtice *et al.* 2019).

## FINANCIAL PRACTICES

Both legal and illegal financial practices have significant health implications. Large corporations, with their greater financial resources, may afford to be involved in the legal aspects of these practices. Wood et al (2023) highlight how financial strategies employed by unhealthy commodity industries generate a ‘double burden’ of maldistribution in LMICs. On one hand, their externalised social and ecological harms disproportionately impact disadvantaged populations and governments. On the other hand, they increasingly transfer wealth and income to a small, privileged elite. Although large corporations typically dominate these industries, many SMEs are also directly or indirectly implicated through their participation in downstream value chains (Reardon *et al.* 2021).

Illegal financial practices such as tax and duty evasion, price fixing, securities and stock market fraud and illicit financial flows are common in LMICs (Global Financial Integrity 2021). These practices, often enabled by political practices and/or poor governance, reduce revenues for the states and disposable income for many, with direct and indirect effects on health and well-being.

Every year, large sums of money are illegally transferred out of LMICs (Global Financial Integrity 2021). These illicit financial flows deplete resources that could be used to fund essential public services, such as health and education. Although such practices occur in all countries and have damaging effects everywhere, their negative impacts are especially pronounced in LMICs due to their limited resources, smaller markets, and inadequate public service structures. Estimates of the scale of these flows are substantial (OECD 2014). For instance, more than $17 billion was syphoned from the financial system in Bangladesh between 2009 and 2024. Much of that money was then transferred out of the country illegally (Travelli and Walid 2024). Depositors were unable to withdraw their savings because the banks did not have enough funds. These inappropriate financial practices had both macro-level effects and micro-level consequences, particularly for depositors, who are mainly low- and middle-income citizens. As a result, individuals were unable to access their cash when they needed it, which hindered their ability to obtain essential goods and services. Although there are no available data on the negative health consequences for depositors, it is possible that some of them were unable to use their funds for necessary purchases and healthcare. Furthermore, the syphoning of money out of the country often occurs in foreign currencies, which then puts pressure on the country’s foreign currency reserve and its ability to purchase goods and services from other countries.

Tax evasion is prevalent in LMICs, particularly among small- and medium-sized businesses. The tax system in these regions is often underdeveloped, creating barriers for taxpayers. Moerenhout and Yang (2022) found that 40% of business owners in Nigeria believe that corruption is common, which negatively affects their tax morale. Additionally, these business owners are often unable to comply with their accounting and tax obligations without paying for professional help, and the costs involved in this, both in terms of time and money, are burdensome for businesses of this size.

There are several examples of stock market manipulation, especially in LMICs, and mainly due to inadequate regulatory frameworks (Azad *et al.* 2014). One common method involves large investors initially making substantial investments to create an artificial bullish trend in the market. Unaware that this is a trap, small investors are drawn into the market. As the stock prices soar to unsustainable levels, disconnected from the underlying fundamentals, the manipulators sell their shares and exit the market. This leaves small investors helpless as they watch their investments lose value and become worthless (Azad *et al.* 2014).

## REPUTATIONAL MANAGEMENT

These practices aim to enhance the legitimacy and credibility of commercial actors, shape their brand image, and reduce risk (Eisenegger and Schranz 2011, Fooks *et al.* 2013). Most often, reputation management practices manifest as corporate social responsibility (CSR), which typically focuses on social, environmental, or public health benefits. These practices often involve voluntary commitments from businesses that claim to offset the harm caused by their core activities (Gilmore *et al.* 2023). CSR serves as a gateway for the commercial sector to establish public–private partnerships with various health and non-health entities in the region. Reputation management activities can be self-initiated or mandated by the laws of the respective countries. For example, an international alcohol company donated a large amount of money to help the Thai government’s 2004 tsunami relief efforts, showcasing a self-initiated effort (Sornpaisarn and Kaewmungkun 2014). It has been alleged that this donation was provided with the intent to gain tax benefits from the government. Conversely, Section 135 of the Companies Act 2013 in India requires every company with a net worth of 5 billion or more Indian rupees, a turnover of 10 billion or more, or a net profit of 50 million or more to allocate at least 2% of their average net profits to CSR initiatives. As a result, India has seen a rise in CSR activities, particularly from the tobacco industry recently (Assunta 2021). For instance, Indian tobacco businesses contributed close to US$37 million to government coffers as part of COVID-19 relief initiatives (Yadav *et al.* 2022). Indeed, commercial actors attempt to utilize the CSR mandates to also improve the credibility and legitimacy of their products. Due to less stable and less developed political systems and economic situations, the reputational management practices in LMICs have been less controlled and monitored than in developed countries.

Many SMEs that produce or market health-harming goods also engage in reputational management—though often in subtler and more resource-constrained ways compared to large corporations. SMEs often frame themselves as ‘local, family-owned, or community-based businesses’ rather than powerful corporate actors (Spence 2016). This narrative softens public perception and shields them from the scrutiny.

## PORTFOLIO OF PRODUCTS

The product portfolios produced or marketed by SMEs span ultra-processed foods, locally produced tobacco (e.g. bidi), alcohol, medicines and hazardous cosmetics, pesticides, antibiotic-intensive aquaculture, plastics and informal retail (Welding *et al.* 2021). Some of the actors operate in an informal market. They normalise harmful products through cultural legitimacy, operate in poorly regulated environments, and sustain health risks. Although these businesses are often small in scale, their collective impact could be substantial. Understanding SMEs’ contributions is, therefore, critical to broadening CDoH analyses beyond multinational corporations and towards the everyday, localised markets where public health harms are embedded.

In recent years, there has been a significant shift in the range of products made available within LMICs. Efforts to prevent noncommunicable diseases go against the business interests of powerful economic operators, as stated by Margaret Chan, the former Director-General of the World Health Organization (WHO 2014b). The literature on CDoH consistently emphasizes noncommunicable diseases. LMICs have experienced a nutrition transition due to trade liberalization and globalization. This shift has led to a significant increase in the consumption of processed foods and items high in fats and sugars, while fruit and vegetable intake has either decreased or remained insufficient (Popkin 2015). The large population and largely untapped markets have created unprecedented opportunities for the commercial sector, especially for businesses that manufacture and market unhealthy products, allowing them to increase their profit margins. A growing proportion of multinational companies has relocated and/or expanded their operations to LMICs and are closely working with SMEs. These changes in trade practices and food consumption patterns have contributed to a rise in the prevalence of non-communicable diseases (Kickbusch *et al.* 2016).

While non-communicable diseases are on the rise, there are numerous examples of unhealthy food production and processing that primarily cause communicable disease outbreaks in LMICs (Grace 2023). The lack of proper storage, the use of unsafe chemicals, food colouring agents, and preservatives, and unhygienic distribution practices are prevalent in LMICs (Grace 2015), were SMEs are the major commercial entities. In recent years, there have been numerous outbreaks of methanol poisoning and fatalities from the consumption of adulterated counterfeit or informally-produced spirit drinks, mainly in LMICs (WHO 2014a). Doreen *et al.* (2020) found that some alcohol sellers adulterated drinking alcohol with methanol to increase profit margins. Some of these practices of production or preservation can cause chronic diseases. For example, in several developing Asian countries, the illegal use of formalin—a highly carcinogenic chemical—as a preservative has been reported in seafood, vegetables, fruits and milk (Kemsawasd *et al.* 2023). While limited financial and technical resources can exacerbate unsafe food practices, the main underlying motive for food fraud is profit-making. Producers and distributors involved opt for cheaper, lower-quality ingredients, harmful additives, or substandard practices to reduce costs and maximize revenue. Often, there is a disregard for public health, as commercial entities push products with little or no concern for safety, quality, or long-term health consequences (Everstine *et al.* 2013). Thus, in the context of LMICs, it would be valid to say that a substantial portion of communicable and non-communicable diseases are attributable to the economic operations of businesses of all sizes—small, medium, and large.

## DISCUSSION

In LMICs, there are widespread CDoH responsible for harming health that are attributed not only to large corporations but also to SMEs. Coupled with inadequate regulations and their enforcement, corruption, and a lack of awareness of health harms from commercial activities or products, LMICs experience a relatively high level of harmful commercial sector practices. The commercial activities discussed in this paper are common in LMICs but are rarely examined through a CDoH lens and are under-represented in the literature. As health care services and social support are limited in LMICs, these adverse determinants are likely to have substantial impact. Thus, alongside addressing the harmful practices of large corporations, it is also important to focus on SMEs. While the adverse health impacts of SMEs may not match those caused by larger entities, SMEs are widespread in LMICs, and, as such, their collective health impacts, though often localised, remain noteworthy. This focus is crucial, especially considering that basic preventive public health measures are often limited in these regions.

Some of the case examples we present above illustrate how different practices are interconnected. For instance, excessive use of pesticides led to a decline in the natural availability of wild-caught fish, which in turn triggered an aggressive push for increased production. This endeavour subsequently was heavily influenced by the marketing of pesticides and antibiotics, ultimately resulting in food that contains harmful chemicals. Similarly, the prominence of market syndicates controlling access to essential products and practices related to the portfolio of products could be intertwined.

It should be noted here that some SMEs are integrated into the supply chains of large corporations (Chen 2019), and this connection may have health consequences. Although such arrangements can be symbiotic for both SMEs and large corporations (Liang *et al.* 2021), there may be power imbalances, which can leave SMEs with little choice but to cut costs in ways that compromise health and safety. The garment sector in South Asian countries is a well-known example: global brands’ relentless pursuit of low prices played a role in poor labour conditions and inadequate building safety, tragically highlighted by the Rana Plaza disaster (Anner 2020). In other sectors, including pharmaceuticals, reliance on small, subcontracted producers for raw ingredients or packaging can result in substandard or falsified products, especially in countries where oversight is limited, with serious implications for patient safety (Mackintosh *et al.* 2023). Taken together, it is therefore important to explore the roles of SMEs through the integrated operational modality.

Many LMICs have democratic systems or at least exhibit democratic elements in which political parties aim to achieve electoral success and consolidate power (DeSilver 2019). To accomplish this, policymakers in LMICs tend to prioritize immediate macroeconomic development metrics, such as GDP, employment growth, and foreign currency reserves, over the health harms from CDoH. They may believe that the benefits from commercial entities, such as tax revenue—which is necessary for fundamental services like education and healthcare—and economic growth outweigh the associated harms (Mukanu *et al.* 2021). Besides, assessing health harms is complex, and their links to commercial entities require longitudinal and multifaceted research. In contrast, macroeconomic indicators are relatively easy to measure and use as tangible evidence of progress. This focus on economic growth creates opportunities for large multinational companies that produce harmful goods and services to expand into LMICs, which often try to attract foreign investment. This expansion is also partly attributed to barriers to operations and marketing of those harmful products in developed countries. Usually, these large companies involve SMEs and individuals to market their products. A notable example of this trend is the expansion of the tobacco industry in LMICs. Additionally, determining the extent or manner of intervention in commercial entities that contribute to health harms is a challenging task, even in high-income countries.

Some of the practices outlined in Table 1 may violate relevant regulations, while others may occur due to insufficient regulations or a lack of enforcement. If certain activities, products, or services comply with national or international rules and regulations, entities engaged in these activities may not be classified as corrupt. However, if they perform their activities, evading the existing rules and regulations, just to gain economic benefits, they can be identified as corrupt. Similarly, within each practice category, the activities vary from ethical to unethical, with a grey zone in between (Gilmore *et al.* 2023). The intersection between adverse CDoH and corruption can be difficult to ascertain and beyond the scope of this paper. The relatively unclear intersection is more problematic in LMICs, where health systems are often under-resourced. In such settings, the power of commercial entities and corrupt practices can disproportionately affect vulnerable populations, leading to poor health outcomes and worsening health inequalities (UNODC and GRACE No date).

The primary aim of CDoH research is to reduce or eliminate the negative impacts arising from commercial activities and enhance the positive aspects and impacts. Achieving this goal extends beyond the health sector and necessitates active participation from multiple other sectors, particularly the business sector. The concept of CDoH aligns with the broader public health initiative of Health in All Policies (Douglas *et al.* 2024). Implementing health in all policies has been challenging, especially in LMICs (Mauti *et al.* 2019), where other sectors often view health as solely the responsibility of the health sector (Hagenaars *et al.* 2024, Islam 2024). Similar obstacles may also hinder the efforts to address adverse CDoH. While there are challenges, the widespread public health harms from CDoH present a significant appeal for improvement. SMEs are vital to the economy in LMICs, and their contributions to local development are widely recognised. The authors acknowledge this vital role and wish to draw attention to the fact that certain commercial practices of some SMEs can be detrimental to public health, and these practices are prevalent in LMICs. Although unlike large commercial entities, SMEs in LMICs generally lack the same structural power or lobbying influence, they can organise into interest groups or peak bodies to present a united front. Yet, most of these harms can be reduced or eliminated through local regulation and enforcement efforts if the individual SMEs or their alliance acknowledge the harmful commercial practices or portfolios and collaborate with public health initiatives.

The establishment of the WHO’s new CDoH unit within the Department of Social Determinants of Health presents an opportunity to raise global awareness about the negative commercial influences on health. The WHO has led and supported numerous public health measures, with the Framework Convention on Tobacco Control (WHO FCTC) serving as a prime example. The ratification of the WHO FCTC has paved the way for the introduction of tobacco control in many LMICs (Puska *et al.* 2019, Spencer *et al.* 2024). Similar treaties could be established for other well-known demerit goods or services, such as alcohol and gambling. However, the negative aspects of CDoH in small- and medium-sized businesses can be attributed to several factors, including inadequate knowledge of the harms, perceived normalcy, lack of alternatives and profit motives. To mitigate the adverse effects of CDoH from these businesses, it may be more effective to implement local initiatives with overarching support from the WHO—particularly in the context of large-scale reductions in WHO resources following the withdrawal of the USA. By enforcing effective public health measures tailored to the local context, most of these issues can be addressed.

The peer-reviewed literature on the CDoH in the context of LMICs, particularly involving SMEs, is limited. Consequently, we had to rely on some grey literature. We recommend conducting both basic and applied research, such as country-level reviews and mapping of CDoH, as well as assessing their impact on health and wellbeing in LMICs. Additionally, we suggest fostering collaborations with authors from LMICs and supporting capacity-building for researchers focused on CDoH in these countries. Finally, we recommend the development of a global forum on CDoH that actively engages collaborators from LMICs.

## Data Availability

All data are incorporated into the article.
